# Self-compassion and work engagement among clinical nurses: the mediating role of moral resilience

**DOI:** 10.3389/fpubh.2025.1507539

**Published:** 2025-01-20

**Authors:** Xingxing Liu, Fang He, Tian Tian, Jun Zhang, Yuanjiao Ji, Yuexia Zhong

**Affiliations:** Outpatient Department, The Second Affiliated Hospital of Air Force Military Medical University, Xi’an, China

**Keywords:** self-compassion, moral resilience, work engagement, mental health, clinical nurses

## Abstract

**Background:**

As the workload of clinical nursing continues to increase, the mental health of nurses has emerged as a critical area of concern. Self-compassion, moral resilience, and work engagement are essential components in enhancing the mental health of clinical nurses. Although it is well-established that self-compassion significantly contributes to improved work engagement, there remains a notable lack of research investigating the specific mechanisms through which self-compassion influences work engagement, particularly from the perspective of moral resilience. This study aimed to address this gap by examining the relationships among self-compassion, moral resilience, and work engagement in clinical nurses, while also validating the mediating role of moral resilience in the relationship between self-compassion and work engagement.

**Methods:**

This study utilized a convenience sampling method to conduct a cross-sectional online survey involving 844 clinical nurses from four tertiary A hospitals in Xi’an, China, between January and March 2024. Participants completed self-report questionnaires that included the Self-Compassion Scale, the Rushton Moral Resilience Scale, and the Utrecht Work Engagement Scale. The data analysis involved descriptive statistics, the Mann–Whitney *U* test, the Kruskal-Wallis *H* rank-sum test, Spearman correlation analysis, and the SPSS PROCESS macro.

**Results:**

A significant positive correlation was observed between clinical nurses’ self-compassion and moral resilience (*r* = 0.700, *p* < 0.01). Additionally, a significant positive correlation was identified between self-compassion and work engagement (*r* = 0.455, *p* < 0.01). Furthermore, there was a significant positive correlation between moral resilience and work engagement (*r* = 0.510, *p* < 0.01). Mediation analysis indicated that moral resilience partially mediates the relationship between clinical nurses’ self-compassion and work engagement. The overall effect of self-compassion on work engagement (*β* = 0.493) consists of both a direct effect (*β* = 0.251) and an indirect effect mediated by moral resilience (*β* = 0.242). Notably, the mediating effect accounts for 49.09% of the total effect.

**Conclusion:**

Clinical nurses’ moral resilience plays a mediating role in the relationship between self-compassion and work engagement. Nursing managers should prioritize fostering and enhancing the self-compassion and moral resilience of clinical nurses to effectively elevate their levels of work engagement. These targeted interventions can ultimately improve not only the mental health and professional well-being of nurses but also the overall quality of care delivered in medical institutions.

## 1 Introduction

The continuous advancement of medical technology, combined with an increasing workload, has resulted in a significant rise in mental health issues among healthcare professionals (1). Previous studies indicated that nurses constitute the largest group within the healthcare workforce, and their mental health challenges were more pronounced compared to those of other professionals (2, 3). Specifically, clinical nurses often experience considerable overwork and stress due to the substantial risks, extensive responsibilities, and high emotional investment associated with their roles. As a result, they are particularly vulnerable to mental health challenges, including anxiety, depression, burnout, and compassion fatigue (4, 5). The repercussions of mental health issues among nurses are far-reaching. These challenges not only correlate with a high incidence of chronic diseases and mental illnesses among nurses (6), but they also adversely affect patient safety and the quality of care provided (7). Moreover, these mental health problems can detrimentally impact nurses’ interpersonal relationships and family life (6). Of particular concern is the potential for these issues to contribute to the loss and shortage of experienced nurses, which could severely compromise the stability of the nursing profession (8). In March 2024, the International Council of Nurses released new mental health nursing guidelines that highlight the importance of nurses’ mental health and well-being, especially in the wake of the COVID−19 pandemic, during which many nurses are still in the process of recovery. Therefore, it is crucial to implement effective strategies to mitigate mental health issues among nurses, thereby safeguarding their well-being and enhancing the quality of nursing care. While the importance of enhancing nurses’ mental health to improve the quality of care is well recognized, there remains a notable lack of comprehensive research investigating the relationships among self-compassion, moral resilience, and work engagement. From a theoretical perspective, a thorough exploration of the relationship between these variables will enrich the theoretical framework of nursing professional psychology, particularly by elucidating the psychological mechanisms that underlie nurses’ professional psychology. Therefore, this study aimed to explore the interconnections among these variables, addressing this research gap and providing new theoretical insights to improve nurses’ mental health and, consequently, the quality of nursing care.

An additional crucial aspect of nursing practice intricately linked to the mental health of nurses is the consideration of ethical issues, with moral distress being the most prevalent and significant among these concerns (9). Nurses frequently encounter institutional, organizational, and situational constraints that hinder their ability to perform nursing actions in accordance with professional norms and ethical standards. This discrepancy between practice and moral principles can result in psychological imbalance and negative emotional experiences for nurses (10). The term “moral distress” describes this phenomenon. Research has shown that ethical challenges contributing to this distress include insufficient professional skills among nurses, difficulties in decision-making, conflicts with healthcare institution policies, and inadequate work infrastructure (11). Additionally, the COVID-19 pandemic has significantly intensified these ethical challenges, leading to an increased incidence of moral distress among nurses (12). A review of relevant research indicates that ethical distress adversely affects both nurses and patients. On one hand, moral distress can lead to heightened stress, decreased job satisfaction, and increased turnover rates among nurses (13). On the other hand, it is also associated with negative mental health outcomes, such as depression, anxiety, and burnout, which can have long-lasting implications (14, 15). Furthermore, moral distress can foster enduring feelings of shame, self-doubt, and guilt among nurses, potentially culminating in the development of moral injury (16). For patients, ethical distress may lead to nurses providing diminished attention and support, ultimately compromising patient health (17). While the moral distress experienced by nurses is often perceived negatively, it is not entirely detrimental. In fact, moral distress can act as a catalyst for nurses to reflect on their own behaviors and integrity. This introspection may foster the development of innovative problem-solving strategies, thereby enhancing the quality of nursing care. Ultimately, this process supports nurses’ mental health and cultivates higher levels of moral resilience (18). Moral resilience refers to an individual’s capacity to maintain or regain their integrity when faced with moral complexity, confusion, pain, or frustration (19). This resilience empowers nurses to persist in upholding moral principles, deepen their moral understanding, and exercise moral courage in confronting ethical challenges. Even when constrained by the medical system and environment, nurses can still strive to act in accordance with their moral principles during ethical dilemmas (20). High levels of moral resilience have been shown to correlate closely with reduced moral distress, lower turnover rates, and decreased job burnout among nurses (9, 21). Most notably, moral resilience is considered a crucial capability for maintaining, restoring, and enhancing nurses’ mental health when they encounter moral distress (22). Consequently, fostering competencies related to moral resilience can assist nurses in mitigating the moral distress they face in the workplace, thereby promoting their mental health, which is vital for improving nursing quality and the overall well-being of nurses.

In recent years, resource factors have attracted increasing scholarly attention, as the various resources available to individuals play a significant role in promoting both physical and mental health (23). Self-compassion is recognized as an essential internal resource and falls within the domain of positive psychology (24). It is defined as the consideration and tolerance an individual demonstrates toward oneself during times of adversity and suffering, as well as the ability to extend to oneself the same kindness and care that one would offer to others (25). Although research on self-compassion is still in its nascent stages, it holds considerable potential for health care applications. There is a growing emphasis on investigating the effects of self-compassion specifically among nurses. A review of the existing literature indicates that self-compassion, as a malleable personal quality, is associated with various positive mental health outcomes and serves as a protective factor for nurses who are vulnerable to work-related stress (26, 27). A cross-sectional study involving Chinese nursing students revealed a negative correlation between self-compassion and levels of anxiety and depression. Moreover, it suggests that interventions designed to enhance self-compassion can effectively mitigate anxiety and depressive symptoms among nursing students (28). Steen’s research demonstrated that self-compassion can significantly reduce anxiety and compassion fatigue among caregivers (29). Numerous studies have affirmed that self-compassion acts as a protective factor against burnout and compassion fatigue in nurses (30–34). Additionally, self-compassion can improve nurses’ capacity to empathize with patients, fostering an awareness and behavior that promotes patient sympathy (35). This is particularly significant in the context of ongoing efforts by countries to enhance the quality of nursing services. Currently, there is a notable absence of direct evidence linking self-compassion to moral resilience in nurses. Existing research indicates that moral resilience is a subset of resilience, with self-compassion serving as a significant predictor of resilience levels among undergraduates. This finding indirectly supports the potential correlation between self-compassion and moral resilience (36). An online survey of war-traumatized veterans demonstrated that self-compassion mediates the relationship between moral injury and suicide (37). Additionally, another online survey conducted in South Korea revealed that self-compassion also plays a mediating role in the relationship between moral injury and depression among adolescents (38). These two studies may offer valuable insights into the connection between self-compassion and moral resilience. Specifically, self-compassion may reduce self-critical tendencies when individuals confront moral harm, thereby enhancing their moral resilience. Theoretically, self-compassion may enhance moral resilience within nursing work environments. By fostering self-compassion, nurses can reduce excessive self-criticism when confronted with ethical dilemmas, such as decisions regarding the allocation of limited medical resources. When nurses practice kindness toward themselves and avoid being overly critical during challenges in moral decision-making or when facing imperfections, they are more likely to uphold their ethical principles, thereby cultivating moral resilience. Consequently, we hypothesized that self-compassion is positively correlated with moral resilience. While research has been conducted on self-compassion and moral resilience, nurses have not been included in these studies. Consequently, there is a significant gap in the literature regarding the relationship between self-compassion and moral resilience among nurses. Self-compassion is recognized as a psychological resource that can enhance individual self-care and may contribute to maintaining psychological balance when nurses encounter moral dilemmas, thereby influencing their moral resilience. The potential relationship between these two constructs could impact nurses’ work engagement; however, this connection has yet to be clearly established.

In the nursing field, work engagement is a concept closely associated with positive psychology and is considered a potential intervention for enhancing nurses’ mental health (39). Emerging from research on occupational burnout, work engagement is characterized as a positive, self-satisfying emotional and cognitive state related to one’s work (40). It emphasizes a strong sense of dedication, absorption, and vigor directed toward professional responsibilities (41). Previous studies have shown that work engagement can mitigate psychological distress, job burnout, and turnover rates among nurses (42, 43), while simultaneously enhancing job satisfaction, work performance, and the overall quality of nursing care (44, 45). Consequently, an increasing number of nursing managers are exploring factors that may enhance nurses’ work engagement, aiming to leverage its positive impact on the quality of nursing services and the healthy development of the nursing profession. Notably, self-compassion and moral resilience have been identified as significant influences on nurses’ work engagement (46, 47). According to existing theories and research findings (46), self-compassion has a positive influence on work engagement. It serves as an internal resource that enables nurses to adopt a tolerant and compassionate attitude toward themselves when confronted with work-related stressors, such as heavy workloads, high-intensity work paces, and misunderstandings from patients. This positive self-attitude not only helps nurses maintain a healthy psychological state but also enhances their commitment to their work. For instance, when a nurse encounters criticism for a minor mistake, a nurse with high self-compassion is less likely to succumb to excessive self-blame; rather, she can swiftly adjust her mindset and continue to engage in her work with renewed enthusiasm. Consequently, we hypothesized that there was a positive relationship between self-compassion and work engagement. Moral toughness is essential for work engagement. Moral resilience enables nurses to maintain professional ethical standards when confronted with complex ethical situations, such as decisions regarding resource allocation, patient privacy protection, and other scenarios involving moral trade-offs (9). This perseverance fosters a sense of professional pride and accomplishment among nurses, thereby enhancing their commitment to their work. Previous research has shown that nurses with high moral resilience are less likely to experience job burnout and turnover intentions when faced with moral dilemmas (21), indirectly illustrating the positive relationship between moral resilience and work engagement. Consequently, we hypothesized a positive correlation between moral resilience and work engagement. According to the Job Demands-Resources (JD-R) model, work engagement is conceptualized as a motivational outcome arising from various types of resources. Individuals with higher levels of personal resources, such as self-compassion and moral resilience, tend to demonstrate greater work engagement (48). Currently, the relationship between self-compassion, moral resilience, and work engagement, as well as the mechanisms underlying these variables, remains unclear within the nursing field. This gap in the literature underscores the significance of the present study. From the perspective of theory construction, clarifying the relationship between these variables will contribute to the development of a more comprehensive professional psychological model for nurses and will enhance relevant theories within the nursing field. Previous research has established a positive correlation between self-compassion and mental health (23). Furthermore, moral resilience has been shown to assist individuals in coping with moral distress (9), thereby supporting mental health, while work engagement is believed to enhance mental health and, consequently, the quality of care provided (39). Therefore, this study focuses on these three variables to analyze their interrelationships and to investigate whether moral resilience serves as a mediating factor between self-compassion and work engagement. This exploration aims to provide theoretical insights for enhancing nurses’ mental health and improving the quality of nursing care, while also contributing new research perspectives within the fields of occupational psychology and nursing.

The theoretical frameworks that informed this study are self-determination theory (SDT) and emotion regulation theory (49, 50). SDT posits that when individuals’ basic psychological needs are satisfied, their intrinsic motivation is enhanced, leading them to invest greater time, energy, creativity, and passion into their work (49). Within the SDT model, self-compassion plays a pivotal role in fulfilling an individual’s psychological needs, thereby enhancing intrinsic motivation and fostering increased work engagement. Conversely, emotion regulation theory explores how individuals manage and adjust their emotions to adapt to their environment and achieve their goals (50). Moral resilience, defined as an individual’s capacity to maintain ethical behavior amidst moral conflict and pressure, is closely linked to emotion regulation. Individuals must effectively manage their emotional responses related to moral decision-making to uphold their moral principles. Furthermore, self-compassion may bolster moral resilience by enabling individuals to cope with moral distress through a non-judgmental form of self-talk. Lastly, work engagement refers to an individual’s emotional commitment and involvement in their work. Building on the previously discussed promotion effect of self-compassion on moral resilience, as well as the relationship between moral resilience and work engagement, we further hypothesized that moral resilience serves as a mediator in the relationship between self-compassion and work engagement. Specifically, when nurses exhibit higher levels of self-compassion, they are better equipped to navigate moral dilemmas, thereby enhancing their moral resilience. This enhancement in moral resilience subsequently fosters a greater degree of work engagement among nurses. For instance, when confronted with the moral dilemma of an unreasonable medical demand from a patient’s family, nurses who possess high self-compassion are more adept at managing their emotions, sustaining their moral resilience, and consequently maintaining a positive level of engagement in their professional responsibilities.

Consequently, our study integrated SDT and emotion regulation theory while reviewing relevant literature and hypothesized a positive correlation between clinical nurses’ self-compassion, moral resilience, and work engagement. Furthermore, we proposed that moral resilience acts as an intermediary factor between self-compassion and work engagement. Our objective was to provide new insights and recommendations for hospital and nursing managers aimed at enhancing nurses’ work engagement and mental health, ultimately serving as a reference for improving nurses’ well-being and the quality of nursing care. Additionally, we aimed for our research to deepen the understanding of the professional psychological mechanisms affecting nurses, thereby offering a novel perspective for both occupational psychology and nursing. This study aimed to investigate the relationship between self-compassion and work engagement among clinical nurses while also examining the mediating role of moral resilience in this relationship. To achieve this, we employed mediation analysis to assess the relationships among clinical nurses’ self-compassion, moral resilience, and work engagement, proposing the following specific hypotheses:

*Hypothesis 1*: Self-compassion positively correlates with work engagement.

*Hypothesis 2*: Moral resilience positively correlates with work engagement.

*Hypothesis 3*: Self-compassion positively correlates with moral resilience.

*Hypothesis 4*: Moral resilience mediates the relationship between self-compassion and work engagement.

## 2 Methods

### 2.1 Study design

From January to March 2024, we utilized a convenience sampling method to conduct a cross-sectional survey among clinical nurses at four comprehensive tertiary-level hospitals situated in Xi’an, Shaanxi Province, in western China.

### 2.2 Participants

The inclusion criteria for the study were as follows: (1) participants must possess professional qualifications and be currently employed; (2) participants must have engaged in clinical nursing practice for a minimum duration of 1 year; and (3) participants must demonstrate an understanding of the study’s content and purpose, as well as cooperate with the investigation. The exclusion criteria were: (1) nurses who were on leave at the time of the survey; (2) nurses who were either practicing or studying within the department under investigation; and (3) nurses who had recently participated in other related research.

### 2.3 Sample size

The study adhered to Kendall’s sample estimation principle (51), which stipulates that the sample size should be 10–20 times the number of variables. In this investigation, the total number of variables was 21, consisting of 8 sociodemographic items and 13 dimensions across three scales. As a result, the calculated sample size range was determined to be between 210 and 420 cases. To account for potential insufficient responses, the original sample size was subsequently increased by 20%, leading to a final required sample size of 252 to 504 cases.

### 2.4 Data collection

To ensure that participating nurses faced no constraints related to time and location, data collection was conducted via an online survey platform. Following the acquisition of informed consent from the nurse management departments of four hospitals, the director of the nursing department at each institution disseminated the link to the electronic questionnaire designed for this study through the nurse management WeChat group. Furthermore, the director provided a detailed explanation regarding the purpose, content, and instructions for completing the questionnaire. Participants answered the questionnaires anonymously, with no identifying information collected about the nurses involved. The first page of the questionnaire functioned as an informed consent form; only those nurses who had read and signed this form were permitted to proceed to the main portion of the questionnaire. Quality control measures were implemented, including: (1) restricting responses to one per IP address to prevent duplicate submissions; (2) setting a time limit for questionnaire completion of approximately 15 to 20 min; and (3) excluding invalid questionnaires that were completed in under 5 min, contained unanswered items, or exhibited identical responses across all items. Ultimately, data were exported from the online survey platform and subjected to analysis following error checking. A total of 868 questionnaires were collected, of which 24 did not meet the quality criteria (15 were completed in less than 5 min, and 9 had missing answers or identical responses). This resulted in 844 valid questionnaires, yielding an effective response rate of 97.24%.

### 2.5 Measurements

#### 2.5.1 Demographic questionnaire

The sociodemographic questionnaire was developed by the researcher after conducting a comprehensive review of the literature. The primary data collected encompassed participants’ gender, age, years of nursing service, education level, professional title, marital status, average monthly income, and employment type. Clinical nurses completed the questionnaire through self-evaluation.

#### 2.5.2 Self-compassion scale

The self-compassion levels of clinical nurses were evaluated using the Self-Compassion Scale (SCS), originally developed by Neff (52) and later translated and revised by Chen (53). The SCS consists of 26 items organized into six dimensions: self-kindness (5 items), self-judgment (5 items), common humanity (4 items), isolation (4 items), mindfulness (4 items), and over-identification (4 items). Example items are “When faced with difficulties, I strive to maintain emotional stability.” and “I exhibit tolerance toward my own flaws and limitations.” Each item is rated on a 5-point Likert scale, ranging from “strongly disagree” to “strongly agree,” with scores assigned from 1 to 5. It is important to note that the dimensions of self-judgment, isolation, and over-identification are reverse scored. The total score on the SCS can range from 26 to 130, with higher scores indicating greater levels of self-compassion. Self-compassion levels were categorized into three tiers based on the average scores of the scale items: low level (1.0 to 2.5 points), medium level (greater than 2.5 to 3.5 points), and high level (greater than 3.5 to 5.0 points). The Cronbach’s *α* coefficient for the total scale was 0.80. Furthermore, the Cronbach’s α coefficients for the six dimensions—self-kindness, common humanity, mindfulness, self-judgment, isolation, and over-identification—were 0.73, 0.70, 0.77, 0.77, 0.77, and 0.70, respectively (54).

#### 2.5.3 Rushton moral resilience scale

The Rushton Moral Resilience Scale was originally developed by Heinze et al. (55) and later translated and revised by Yang et al. (56) to produce the Chinese version of the Rushton Moral Resilience Scale (C-RMRS), specifically designed to assess the moral resilience of clinical nurses. The C-RMRS comprises four dimensions and consists of a total of 16 items: four items focus on responses to moral adversity, four items pertain to moral efficacy, five items address relationship integrity, and the remaining three items concentrate on personal integrity. Example items are “I often feel a sense of powerlessness when confronted with challenging ethical dilemmas.” and “My actions consistently align with my values.” Each item is evaluated using a 4-point Likert scale, with responses ranging from 1 (disagree) to 4 (agree), and 11 items are reverse scored. The total score for the C-RMRS ranges from 16 to 64, with higher scores indicating greater levels of moral resilience. The Cronbach’s *α* coefficient for the total scale was 0.76. The coefficients for the four dimensions—response to moral adversity, personal integrity, moral efficacy, and relationship integrity—were 0.842, 0.523, 0.796, and 0.820, respectively. C-RMRS demonstrated good reliability and validity and has been effectively validated among Chinese nurses (57).

#### 2.5.4 Utrecht work engagement scale

The Utrecht Work Engagement Scale was initially developed by Schaufeli et al. (58) and later translated, revised, and validated by Li et al. (59), leading to the creation of the Chinese version known as the Chinese Utrecht Work Engagement Scale (C-UWES). The C-UWES consists of a total of 9 items, which are organized into three dimensions: vigor, dedication, and absorption, with each dimension containing 3 items. Example items are “I am deeply passionate about my work.” and “I take great pride in the work that I undertake.” Each item is evaluated using a 7-point scale, ranging from 0 (never) to 6 (always). The overall C-UWES score can vary from 0 to 54, with higher scores indicating a greater level of individual work engagement. Work engagement levels are categorized based on the average item scores: scores of ≤2 points signify low engagement, scores of ≥4 points denote high engagement, and scores greater than 2 but less than 4 indicate medium engagement. The Cronbach’s *α* value for the total scale was 0.93, while the Cronbach’s *α* coefficients for the three dimensions—vigor, dedication, and absorption—were 0.78, 0.80, and 0.81, respectively.

### 2.6 Ethical considerations

The study was conducted in compliance with the Declaration of Helsinki and received approval from the Ethics Committee of the Second Affiliated Hospital of Air Force Medical University in Xi’an, Shaanxi Province, China. This research upheld the principle of voluntary participation, with all participants providing informed consent. Participants were informed of their right to refuse or withdraw from the study at any point without incurring any loss of benefits. All data generated from the questionnaire will be stored confidentially and will be accessible solely to the researchers.

### 2.7 Data analysis

Statistical analysis was performed using SPSS version 25.0 and the PROCESS macro (version 3.3). Initially, a normality test was conducted, with measurement data presented as medians and interquartile ranges, while count data was expressed in terms of frequencies and percentages. These descriptive statistics facilitated the analysis of the sociodemographic characteristics of the participating nurses, as well as their scores on self-compassion, moral resilience, and work engagement. Subsequently, the Mann–Whitney *U* test and the Kruskal–Wallis *H* rank-sum test were employed to compare differences in work engagement across the sociodemographic characteristics of the participants. Following this, Spearman correlation analysis was conducted to examine the relationships among the three variables: self-compassion, moral resilience, and work engagement. Finally, this study utilized PROCESS Model 4 to investigate the mediating role of moral resilience between self-compassion and work engagement among clinical nurses while controlling for all statistically significant covariates identified in the sociodemographic analysis (60). This method relies on ordinary least squares regression and the bias-adjusted bootstrapping technique. Additionally, to evaluate the impact of self-compassion on clinical nurses’ work engagement, bias-corrected percentile bootstrap distributions with 95% confidence intervals were calculated from 5,000 bootstrap samples (60). All statistical tests were two-tailed, and the significance level was set at *p* < 0.05 to determine significant differences.

## 3 Results

### 3.1 Common method bias tests

This study conducted a Harman single-factor test on all scale items. The results indicated that the first factor accounted for only 21.05% of the total variance, which is significantly lower than the critical threshold of 40% (61). This finding suggests that the study did not exhibit serious common method deviation (61).

### 3.2 Participants’ demographic characteristics and their distribution by work engagement scores

Table 1 presents the sociodemographic characteristics of the 844 participants. A significant majority of the participants were female (777, 92.06%). The ages of the participants ranged from 25 to 49 years, with the largest group being those aged 30–40 years (606 individuals, 71.80%). The years of nursing service varied from 2 to 25 years, with the majority of participants (368 individuals, 43.60%) having between 11 and 15 years of nursing experience. Most participants held a bachelor’s degree (777, 92.06%). Additionally, a significant portion of the participants (388, 45.97%) held the title of Nurse Practitioner. A substantial percentage of participants (79.27%) were married. Furthermore, more than 50% of participants reported an average monthly income ranging from 5,001 to 10,000 RMB (approximately 709.24 to 1418.19 US dollars). Similarly, over 50% of the participants were employed under a contract system.

**Table 1:** Participants’ demographic characteristics and their distribution by work engagement scores (*n* = 844).

Characteristics	*N* (%)	Work engagement		
		Median (P25, P75)	*Z/H*	*P*
Gender				
Male	67(7.94)	36.00(36.00,42.00)	−1.781^a^	0.075
Female	777(92.06)	37.00(36.00,40.00)		
Age (years)				
<30	153(18.13)	36.00(36.00,40.00)	3.130^b^	0.209
30 ~ 40	606(71.80)	37.00(36.00,40.00)		
>40	85(10.07)	37.00(34.00,45.00)		
Length of service in nursing (years)				
≤5	164(19.43)	36.00(35.00,39.00)	27.896^b^	<0.001
6 ~ 10	220(26.07)	36.00(36.00,39.00)		
11 ~ 15	368(43.60)	37.00(36.00,41.00)		
>15	92(10.90)	38.00(36.00,45.00)		
Educational level				
College or below	38(4.50)	37.00(32.00,45.00)	1.737^b^	0.420
Undergraduate	777(92.06)	36.00(36.00,40.00)		
Postgraduate or above	29(3.44)	38.00(34.00,3.00)		
Professional title				
The nurse	232(27.49)	37.00(2.00,40.00)	35.067^b^	<0.001
Nurse practitioner	388(45.97)	36.00(36.00,39.00)		
Nurse-in-charge	170(20.14)	38.00(36.00,42.00)		
Deputy director nurse or above	54(6.40)	39.00(36.00,42.00)		
Marital status				
Unmarried	175(20.73)	36.00(35.00,40.00)	−2.259^a^	0.024
Married	669(79.27)	37.00(36.00,41.00)		
Average monthly income (RMB)				
≤5,000	93(11.02)	36.00(35.00,39.00)	4.461^b^	0.107
5,001 ~ 10,000	588(69.67)	37.00(36.00,40.00)		
10,001 ~ 15,000	163(19.31)	36.00(36.00,42.00)		
Employment type				
Officially on staff	29(3.44)	45.00(36.00,45.00)	14.972^b^	0.001
Contract system	744(88.15)	37.00(36.00,40.00)		
Personnel agent	71(8.41)	36.00(32.00,38.00)		

^aZ, bH.^

Significant differences were observed in the work engagement scores of 844 clinical nurses based on four demographic factors: years of nursing service, professional title, marital status, and employment type (*p* < 0.05) (Table 1).

### 3.3 Descriptive statistical analysis of variable scores

The median values for the SCS, C-RMRS, and C-UWES were 88.00, 45.00, and 37.00, respectively. Table 2 presents the results of the descriptive statistical analysis for these variable scores.

**Table 2:** Descriptive statistical analysis of variable scores (*n* = 844).

Variables	Items	Minimum	Maximum	Median (P25, P75)
Self-compassion	26	67.00	121.00	88.00(80.00,97.00)
Self-kindness	5	11.00	25.00	17.00(16.00,20.00)
Self-judgment	5	5.00	24.00	18.00(15.00,19.00)
Common humanity	4	7.00	20.00	13.00(12.00,15.00)
Isolation	4	4.00	20.00	13.00(12.00,15.00)
Mindfulness	4	9.00	20.00	14.00(12.00,16.00)
Over-identification	4	4.00	17.00	13.00(12.00,15.00)
Moral resilience	16	30.00	61.00	45.00(39.25,48.00)
Response to moral adversity	4	4.00	16.00	11.00(9.00,13.00)
Moral efficacy	4	7.00	16.00	12.00(11.00,14.00)
Relationship integrity	5	7.00	20.00	14.00(11.00,15.00)
Personal integrity	3	4.00	10.00	7.00(7.00,8.00)
Work engagement	9	29.00	45.00	37.00(36.00,40.00)
Vigor	3	10.00	15.00	13.00(12.00,14.00)
Dedication	3	6.00	15.00	12.00(11.00,12.00)
Absorption	3	10.00	15.00	12.00(12.00,14.00)

### 3.4 Correlations of the study variables

Table 3 illustrates the correlations among self-compassion, moral resilience, and work engagement. The results of the Spearman correlation analysis revealed a strong positive correlation between self-compassion and moral resilience (*r* = 0.700, *p* < 0.01). Furthermore, a positive correlation was observed between self-compassion and work engagement (*r* = 0.455, *p* < 0.01), as well as between moral resilience and work engagement (*r* = 0.510, *p* < 0.01).

**Table 3:** Correlations of the study variables (*n* = 844).

Variables	1	2	3	4	5	6	7	8	9	10	11	12	13	14	15	16
1	1.000															
2	0.660^**^	1.000														
3	0.667^**^	0.150^**^	1.000													
4	0.249^**^	0.537^**^	−0.315^**^	1.000												
5	0.633^**^	0.058	0.616^**^	−0.137^**^	1.000											
6	0.670^**^	0.719^**^	0.135^**^	0.504^**^	0.155^**^	1.000										
7	0.601^**^	0.100^**^	0.622^**^	−0.375^**^	0.655^**^	0.158^**^	1.000									
8	0.700^**^	0.417^**^	0.435^**^	0.134^**^	0.583^**^	0.527^**^	0.455^**^	1.000								
9	0.605^**^	0.422^**^	0.386^**^	0.042	0.464^**^	0.466^**^	0.434^**^	0.759^**^	1.000							
10	0.321^**^	0.167^**^	0.150^**^	0.146^**^	0.275^**^	0.295^**^	0.028	0.529^**^	0.139^**^	1.000						
11	0.538^**^	0.316^**^	0.391^**^	0.111^**^	0.449^**^	0.384^**^	0.357^**^	0.785^**^	0.459^**^	0.265^**^	1.000					
12	0.271^**^	0.166^**^	0.102^**^	0.121^**^	0.263^**^	0.178^**^	0.292^**^	0.374^**^	0.295^**^	−0.094^**^	0.274^**^	1.000				
13	0.455^**^	0.346^**^	0.182^**^	0.194^**^	0.217^**^	0.398^**^	0.244^**^	0.510^**^	0.251^**^	0.520^**^	0.388^**^	0.081^*^	1.000			
14	0.456^**^	0.321^**^	0.215^**^	0.162^**^	0.231^**^	0.394^**^	0.276^**^	0.534^**^	0.285^**^	0.565^**^	0.386^**^	0.011	0.889^**^	1.000		
15	0.298^**^	0.299^**^	0.091^**^	0.202^**^	0.092^**^	0.187^**^	0.170^**^	0.258^**^	0.089^*^	0.224^**^	0.202^**^	0.161^**^	0.788^**^	0.560^**^	1.000	
16	0.491^**^	0.351^**^	0.239^**^	0.127^**^	0.259^**^	0.471^**^	0.239^**^	0.597^**^	0.383^**^	0.533^**^	0.479^**^	0.108^**^	0.846^**^	0.788^**^	0.461^**^	1.000

*P < 0.05, **P < 0.01, 1 = self-compassion; 2 = self-kindness; 3 = self-judgment; 4 = common humanity; 5 = isolation; 6 = mindfulness; 7 = over-identification; 8 = moral resilience; 9 = response to moral adversity; 10 = moral efficacy; 11 = relationship integrity; 12 = personal integrity; 13 = work engagement; 14 = vigor; 15 = dedication; 16 = absorption.

### 3.5 Mediating effect of moral resilience between self-compassion and work engagement

We employed the PROCESS macro (version 3.3) to conduct a bootstrapping analysis that examined the mediating role of moral resilience in the relationship between self-compassion and work engagement while controlling for potential confounding variables. The purpose of controlling for confounding variables was to reduce their potential interference with the research outcomes, thereby facilitating a more precise evaluation of the relationship between self-compassion and work engagement. The analysis of these confounding variables utilized the Mann–Whitney *U* test and the Kruskal–Wallis *H* rank-sum test, treating sociodemographic variables as independent variables and work engagement as the dependent variable. The control variables in the model included four factors: years of nursing service, professional title, marital status, and employment type. This selection was guided by the statistically significant differences observed in work engagement scores across these factors (*p* < 0.05) (see Tables 1, 4).

**Table 4:** The mediating model of moral resilience between self-compassion and work engagement (*n* = 844).

Outcome variable	Predictor variable	*R*	*R^2^*	*F*(df)	*β*	*t*
Work engagement						
	Self-compassion	0541	0.293	69.286	0.493	16.296^***^
	Length of service in nursing				0.137	3.163^**^
	Professional title				0.139	3.594^***^
	Marital status				−0.345	−3.639^***^
	Employment type				−0.331	−3.517^***^
Moral resilience						
	Self-compassion	0.744	0.553	207.409	0.705	29.297^***^
	Length of service in nursing				0.070	2.048^*^
	Professional title				−0.111	−3.613^***^
	Marital status				−0.155	−2.056^*^
	Employment type				−0.707	−9.459^***^
Work engagement						
	Self-compassion	0.588	0.345	73.560	0.251	6.057^***^
	Moral resilience				0.344	8.213^***^
	Length of service in nursing				0.112	2.698^**^
	Professional title				0.178	4.723^***^
	Marital status				−0.291	−3.190^**^
	Employment type				−0.088	−0.922

^*^P < 0.05, ^**^P < 0.01, ^***^P < 0.001.

Table 4 presents the results of testing the mediating effect using 5,000 bootstrap samples. The findings indicated that self-compassion significantly predicted work engagement. Notably, even after accounting for moral resilience, the direct predictive effect of self-compassion on work engagement remained significant. The results further demonstrated that both self-compassion and moral resilience were significant predictors of work engagement (*β* = 0.493, *p* < 0.001; *β* = 0.344, *p* < 0.001). Importantly, the effect of self-compassion on work engagement was diminished in the model that included moral resilience (*β* = 0.251, *p* < 0.001). Additionally, the study identified a significant association between self-compassion and moral resilience (*β* = 0.705, *p* < 0.001). These findings suggest that moral resilience serves as a partial mediator in the relationship between self-compassion and work engagement.

**Figure 1:**
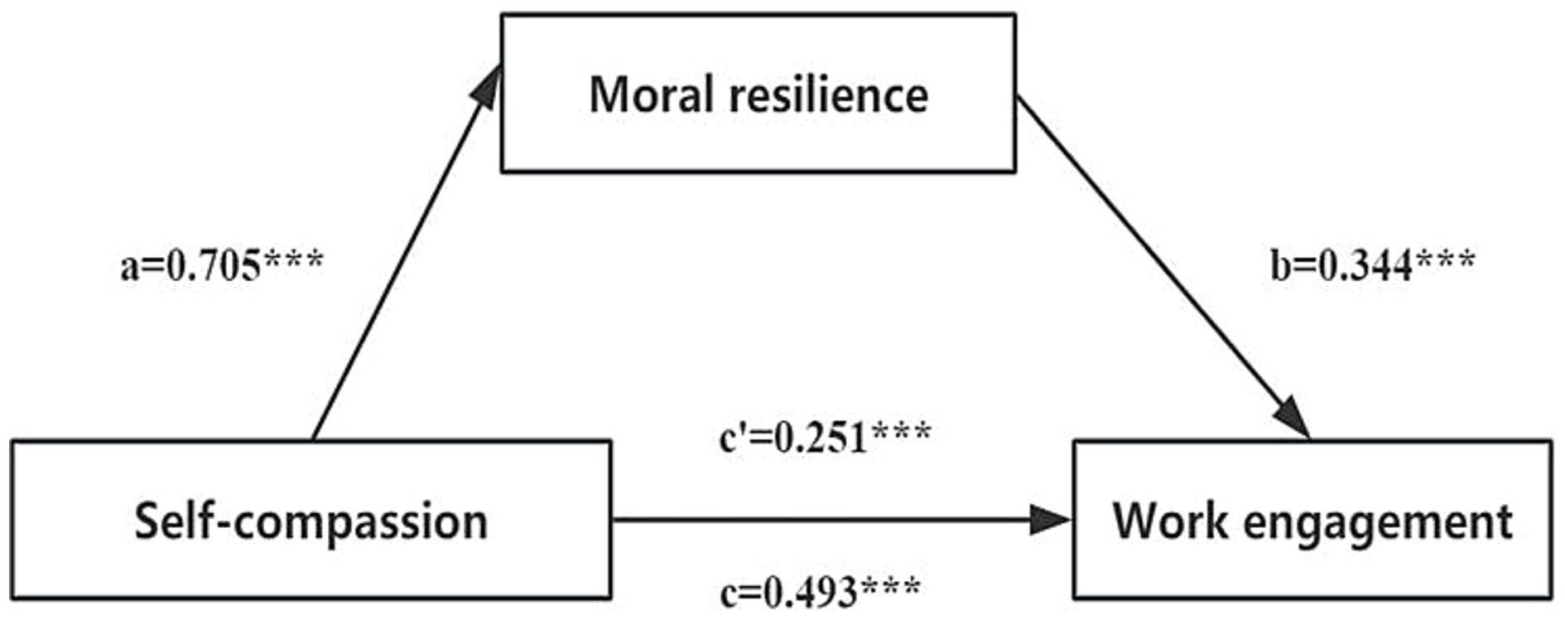
The mediating effect model of clinical nurses’ moral resilience between self-compassion and work engagement. a, the effect of self-compassion on moral resilience; b, the effect of moral resilience on work engagement; c, the total effect of self-compassion on work engagement; c’, the direct effect of self-compassion on work engagement; ab, the mediating effect of moral resilience. ^***^p < 0.001.

The study revealed that the 95% bootstrap confidence intervals for the direct effect of self-compassion on work engagement, as well as the mediating effect of moral resilience, did not include zero (95% Boot CI = [0.170, 0.333]; [0.176, 0.310], refer to Table 5). This finding indicates that self-compassion can indirectly predict work engagement through the mediating effect of moral resilience. The relationship between these variables is illustrated in Figure 1, where the direct effect is 0.251 and the mediation effect is 0.242. Notably, the mediating effect accounts for 49.09% of the total effect.

**Table 5:** Decomposition table of total effect, direct effect, and mediating effect (*n* = 844).

	Effect	Boot SE	95% Boot LLCI	95% Boot ULCI	The relative effect (%)
Total effect	0.493^***^	0.030	0.434	0.553	
Direct effect	0.251^***^	0.042	0.170	0.333	50.91
Mediating effect	0.242^***^	0.034	0.176	0.310	49.09

Boot SE, bootstrap standard error; 95% Boot LLCI, the lowest level of the 95% Bootstrap confidence interval; 95% Boot ULCI, the highest level of the 95% Bootstrap confidence interval.

^***^p < 0.001.

## 4 Discussion

This study aimed to investigate the relationship between self-compassion and work engagement among clinical nurses while also examining the mediating role of moral resilience in this relationship. The findings are intended to offer valuable insights for improving work engagement and mental health in this population. Our results revealed a significant positive correlation among self-compassion, moral resilience, and work engagement. Importantly, moral resilience was identified as a mediator between self-compassion and work engagement in clinical nurses. This suggests that self-compassion may help nurses cope more effectively with the moral distress they face. By fostering moral resilience, nurses are likely to maintain a positive outlook and sustain their engagement in work. Notably, this study innovatively combined SDT and emotion regulation theory to explore the interactions among self-compassion, moral resilience, and work engagement in clinical nurses, thereby clarifying the specific mediating role of moral resilience. This research provides a scientific basis for interventions aimed at enhancing the mental health of clinical nurses. Ultimately, this study enhances researchers’ understanding of the professional psychological mechanisms that influence nurses.

The SCS scores of clinical nurses were found to be at a medium level, which is higher than the results reported in Yang’s study (62). This finding suggests that while the level of self-compassion among clinical nurses has improved compared to previous assessments, there remains significant potential for further enhancement. The observed difference may be attributed to the varying distribution of years of nursing experience among participants. In this study, senior clinical nurses with over 10 years of experience comprised 54.50% of the sample, indicating that they possess more extensive life and clinical experiences. When confronted with challenges or nursing errors, these senior nurses tend not to blame themselves or excessively dwell on their reflections. Instead, they maintain a clear mindset and utilize their clinical practice experience and technical skills to effectively address practical difficulties. As a result, senior clinical nurses demonstrate higher levels of self-compassion, particularly in terms of self-kindness and mindfulness regulation. Germer’s research (63) indicates that self-compassion can be enhanced through targeted training. Therefore, it is recommended that nursing managers organize relevant educational programs to assist clinical nurses in recognizing and accepting their emotions, especially the negative feelings encountered in the workplace, and to cultivate self-compassion skills. Furthermore, fostering a supportive environment that encourages nurses to share their challenges and feelings at work can further enhance their self-compassion abilities.

Moral resilience is a critical component of clinical nurses’ careers, enabling them to maintain professional conduct and sound decision-making when faced with ethical challenges and distress (19). This resilience is vital for delivering high-quality patient care, upholding professional standards, and promoting nurse well-being. The C-RMRS scores of clinical nurses in this study were lower than those reported by Hu et al. (64) for ICU nurses. This difference may be attributed to the distinct working environments of clinical nurses compared to their ICU counterparts. Patients in the intensive care unit are typically critically ill, experience rapid fluctuations in their condition, and face a heightened risk of mortality. As a result, ICU nurses often endure greater work-related stress and emotional burdens than clinical nurses. This high-pressure setting necessitates that ICU nurses possess considerable moral fortitude to effectively navigate the ethical dilemmas involving patients and their families. With the evolution of medical models and the incorporation of new technologies in nursing practice, the ethical dilemmas encountered by nurses in clinical settings have become increasingly complex (17). Therefore, it is imperative for clinical nurses to continuously enhance their moral resilience to effectively manage these challenges. This highlights the necessity for nursing managers to offer additional support to clinical nurses, including opportunities for practical training on ethical dilemmas, the development of coping strategies, the cultivation of a positive ethical climate within healthcare facilities, and the enhancement of nurses’ professional self-concept. By implementing these strategies, nurses will be better equipped to address the ethical challenges they face in their work, thereby strengthening their moral resilience.

In comparison to the findings reported in the Iranian study (46), the clinical nurses’ work engagement scores in this study were higher; however, they were lower than the results of another study conducted in Beijing, China (65). This discrepancy may be attributed to varying levels of regional economic development and the timing of the surveys. Notably, China’s overall economic development is generally more advanced than that of Iran, with Beijing’s economic standing significantly surpassing that of the region examined in this study. In more economically developed areas, nurses typically receive higher salaries, which contributes to enhanced professional happiness and satisfaction. Furthermore, improved economic conditions enable hospitals to offer better working environments for nurses, thereby alleviating their workload, increasing work efficiency, and subsequently enhancing work engagement. Additionally, it is important to consider that the Iranian study was conducted during the COVID-19 pandemic, a period marked by high turnover rates and substantial workloads for nurses, which significantly diminished their work efficiency and engagement (13). Lastly, our research findings indicate that factors such as clinical nurses’ years of service, professional titles, marital status, and employment type significantly impact their levels of work engagement. Specifically, greater years of service and higher professional titles correlate with increased clinical nursing experience, leading to heightened feelings of accomplishment and satisfaction. This intrinsic motivation fosters greater engagement among nurses in their work (66). Married nurses often benefit from the emotional and life support provided by their spouses, which enhances their ability to manage work-related stress and allows for a greater focus on their professional responsibilities (66). In China, formally employed nurses generally experience greater job stability than their contract and temporary counterparts. This job security can enhance their commitment to their roles. Moreover, formally employed nurses benefit from improved economic incentives and social support, which contribute to elevated job satisfaction—a critical factor in fostering work engagement. Based on these findings, it is recommended that nursing managers focus on helping nurses develop their professional skills and clarify pathways for career advancement. Additionally, the implementation of a fair performance appraisal system that rewards employees based on their work performance is crucial. Furthermore, providing essential emotional and social support can mitigate nurses’ stress, thereby boosting their enthusiasm for work engagement.

Self-compassion has emerged as a critical factor in enhancing work engagement (47). Research demonstrates a significant and positive correlation between self-compassion and work engagement. Our first hypothesis was validated, consistent with previous studies involving physicians and emergency department nurses (46, 67). During the COVID-19 pandemic, Iranian scholars conducted a survey study involving 424 ICU nurses across three hospitals (46). The findings indicated that work engagement, mental health, and work performance were all positively correlated with self-compassion. Additionally, a study conducted in Canada by Babenko et al. (67) confirmed that self-compassionate doctors exhibited higher levels of positive work engagement compared to their less self-compassionate counterparts. These results provide valuable insights into the relationship between self-compassion and work engagement, thereby supporting the related hypotheses. Additionally, self-compassion is viewed as a constructive development of personal resources (24). A potential explanation, rooted in resource conservation theory, posits that self-compassion may help individuals maintain a healthier state of psychological resource conservation, thereby enabling them to acquire additional resources through increased work input (68). Several studies (28, 30, 32) have shown that self-compassion can reduce professional burnout among nurses by alleviating negative emotions, which is vital for enhancing their work engagement. This indicates that self-compassion is closely linked to the emotional regulation mechanisms of nurses; by activating these mechanisms, it promotes better mental health and ultimately enhances work engagement. Furthermore, findings from a systematic review (69) support the indirect role of emotional regulation in the relationship between self-care and mental health, reinforcing the conclusions of this study. Collectively, these findings highlight the importance of self-compassion interventions, as self-compassion is a highly malleable trait that serves as a significant moderator of clinical nurses’ work engagement. Recent research has confirmed that intervention methods, such as mindfulness self-compassion education (70), self-compassion letter tools (71), and pet ownership (54), positively influence the enhancement of self-compassion among nurses. It is recommended that nursing managers effectively implement these strategies to foster nurses’ work engagement by enhancing their self-compassion skills.

From the perspective of positive psychology, moral resilience is defined as the positive psychological attributes that individuals exhibit when faced with moral distress and challenges. This resilience empowers individuals to uphold their core values and moral principles while fully dedicating themselves to their work. Research findings reveal a significant positive correlation between moral resilience and work engagement, thereby substantiating our second hypothesis. A cross-sectional study of emergency nurses conducted by Clark et al. (47) provided evidence supporting our hypothesis. While the study examined the relationship between resilience, moral dilemmas, and workplace engagement, it found that higher adaptability among nurses—which can be viewed as a related concept to moral resilience—correlates with increased workplace engagement, a factor closely associated with overall work engagement. This finding reinforces the conclusion of our study that moral resilience is positively related to work engagement. According to Rushton (72), nurses’ capacities to manage moral adversity in clinical practice can be bolstered by fostering aspects of moral resilience, such as moral confidence and moral competence. This enhancement can contribute to the establishment of a healthy work environment and a reduction in nurse turnover rates, ultimately leading to increased work engagement. A web-based survey study involving emergency department nurses concluded that workplace engagement could be improved through interventions designed to alleviate moral distress (47). Furthermore, prior research has confirmed that moral resilience serves a protective function against moral distress, burnout, and turnover intention (21). Consequently, for nurses working in high-pressure environments, promoting moral resilience is advantageous for enhancing their adaptability and engagement. It is advisable for nursing managers to broaden and deepen ethics education, ensuring its integration throughout the entire nursing career cycle to promote sustainability. Equally important is the necessity to foster an ethical environment that supports the practical application of this education. By implementing these strategies, the moral resilience of clinical nurses can be consistently enhanced, resulting in increased work engagement.

The research findings indicate a positive relationship between self-compassion and moral resilience, thereby supporting our third hypothesis. This correlation suggests that nurses with higher levels of self-compassion are more likely to exhibit stronger moral resilience. A cross-sectional study of nursing students in Iran identified self-compassion as a predictor of moral intelligence (73). Both moral resilience and moral intelligence represent competencies that individuals demonstrate when faced with moral challenges and dilemmas, which involve their judgment in moral decision-making and actions. Furthermore, self-compassion serves as a supportive mechanism for mental health, providing psychological buffering and protection. This support enables individuals to maintain clarity in judgment and decision-making when confronted with moral challenges, thereby enhancing adherence to moral principles. Jang (38) investigated 1,567 Korean adolescents and found that self-compassion serves as a mediating factor in the relationship between moral injury and depression. This suggests that self-compassion significantly influences an individual’s psychological state when confronted with morally related challenges, such as moral injury. Similarly, when nurses encounter moral dilemmas, self-compassion can function as a supportive mechanism for mental health, paralleling its role for adolescents facing moral injury. This quality can assist individuals in maintaining a positive mental state during moral challenges, thereby enhancing moral resilience. These findings further emphasize the positive relationship between self-compassion and moral resilience. According to resource conservation theory (68), the moral dilemmas and emotional labor experienced by nurses can deplete their resources; however, self-compassion may mitigate this depletion, thus preserving moral judgment and behavior while enhancing moral resilience. Consequently, nursing managers should acknowledge the beneficial role of self-compassion in strengthening the moral resilience of clinical nurses and implement effective interventions aimed at enhancing self-compassion levels.

Mediation effect analysis revealed that the moral resilience of clinical nurses significantly mediates the relationship between self-compassion and work engagement, with a mediating effect ratio of 49.09%. This finding supports our fourth hypothesis, indicating that self-compassion not only directly predicts work engagement but also enhances it by fostering moral resilience. According to SDT (49), the satisfaction of intrinsic motivation and basic psychological needs is linked to an individual’s positive psychological state and behavioral investment. As a source of intrinsic motivation, self-compassion helps clinical nurses maintain positive self-evaluation and emotional regulation when faced with workplace pressures and moral challenges, thereby enhancing work engagement. In this context, affective mediation theory provides insights into how nurses manage their emotions to adapt to their environments (50). Furthermore, moral resilience plays a crucial mediating role in this process, as it relates to how individuals uphold their values and beliefs in the face of adversity, aligning with the fundamental aspects of self-compassion. Moral resilience may further enhance work engagement by offering a form of psychological toughness that enables nurses to maintain emotional stability. In Li et al.’s (9) study on pediatric nurses, moral toughness was identified as a moderating factor between nurses’ moral distress and job embeddedness. This finding is relevant to our research. While Li’s study focused on moral distress and job embeddedness, our investigation explores the relationship between self-compassion, moral resilience, and work engagement. Both studies underscore the significant role of moral resilience in nursing practice. Specifically, our research posits that self-compassion can enhance moral resilience, thereby improving work engagement. This aligns with the literature that emphasizes the influence of moral resilience on work-related factors among nurses. Furthermore, it suggests that moral resilience mediates the relationship between self-compassion and work engagement, providing supporting evidence for this connection. In summary, self-compassion aids nurses in effectively navigating ethical challenges and emotional stress at work by fostering moral resilience, which in turn increases their work engagement.

## 5 Implication for nursing management

The most striking finding of our study was that moral resilience played a mediating role in the relationship between self-compassion and work engagement among clinical nurses. This finding has significant theoretical and practical implications. Theoretically, it further validates the complex relationship model among self-compassion, moral resilience, and work engagement, thereby providing a foundation for the refined development of nursing professional psychology theory. Practically, our research conclusions offer specific and actionable insights for enhancing clinical nurses’ work engagement and mental health, ultimately guiding efforts to improve their overall well-being and the quality of nursing care. For instance, nursing managers can develop targeted strategies to enhance nurses’ self-compassion and moral resilience based on our research findings, such as implementing specialized training programs. Furthermore, our research deepens the understanding of the psychological mechanisms inherent in the nursing profession, presenting a novel perspective on nursing professional psychology. Nursing managers should recognize the critical role of self-compassion and moral resilience in fostering nurses’ work engagement. Administrators are encouraged to invest in nurses’ personal development by providing essential resources and training that facilitate the cultivation of greater self-compassion and moral resilience. Concurrently, managers should actively monitor the work environment to promptly identify and address factors that may lead to ethical dilemmas, thereby safeguarding nurses’ mental health and professional well-being. By implementing these strategies, care managers can contribute to the formation of a healthier and more efficient care team.

## 6 Limitations

Several limitations must be considered when interpreting the results. Firstly, to facilitate participation without restrictions related to time and location, we employed an online questionnaire tool and did not provide face-to-face explanations of the questionnaire’s contents to participants. This approach may have influenced the research findings. Secondly, the cross-sectional design constrains our ability to establish causal relationships between the variables, highlighting the need for further longitudinal studies to verify these relationships. Thirdly, this study utilized convenience sampling, with all participants drawn from tertiary hospitals in Xi’an. Future research should aim to broaden the geographic scope, types of hospitals, and sample sizes. Fourthly, the data collection relied on self-reports from clinical nurses, which could introduce expectation bias. Lastly, this study did not perform a confirmatory factor analysis, which may affect the generalizability of the research findings. Despite these limitations, our study is the first to investigate the relationship between self-compassion, moral resilience, and work engagement among clinical nurses, offering a novel perspective on understanding nurses’ mental health and work engagement. Additionally, the research reflects an integration of interdisciplinary concepts, including psychology and ethics.

## 7 Conclusion

This study revealed the mediating effect of clinical nurses’ moral resilience on the relationship between self-compassion and work engagement. This finding has significant theoretical and practical implications. Theoretically, it introduces new elements to the existing literature on nurses’ mental health, thereby contributing to the development of a more comprehensive professional psychological framework for nurses. Practically, it offers a fresh perspective on understanding the dynamic mechanisms underlying nurses’ mental health. Our results have important implications for clinical care management practices. Nursing managers should prioritize the cultivation of self-compassion and moral resilience among clinical nurses. By implementing self-compassion training and moral resilience enhancement projects with specific objectives, targeted interventions should be developed based on the findings of this study. Such initiatives aim to assist nurses in establishing a healthy professional psychological state, ultimately enhancing their work engagement and the overall quality of care. Additionally, we encourage medical institutions to place a greater emphasis on the professional mental health of nurses and to collaboratively foster a healthier and more supportive working environment through comprehensive support and intervention strategies, ultimately promoting the holistic personal and professional development of nurses.

## Data Availability

The original contributions presented in the study are included in the article/Supplementary material, further inquiries can be directed to the corresponding author.
